# Laboratory performance of genome-wide cfDNA for copy number variants as compared to prenatal microarray

**DOI:** 10.1186/s13039-023-00642-4

**Published:** 2023-06-10

**Authors:** Erica Soster, John Tynan, Clare Gibbons, Wendy Meschino, Jenna Wardrop, Eyad Almasri, Stuart Schwartz, Graham McLennan

**Affiliations:** 1 Labcorp®, La Jolla, CA USA; 2 Genetics Program, North York General Hospital, Toronto, ON Canada; 3 Department of Paediatrics, University of Toronto, Toronto, ON Canada; 4 Labcorp®, Research Triangle Park, NC USA; 5 Present Address: PetDx, The Center for Novel Therapeutics, La Jolla, CA USA

**Keywords:** Non-invasive prenatal testing (NIPT), Prenatal screening, Circulating cell-free DNA (cfDNA), Genome-wide NIPT, Prenatal diagnosis, Rare aneuploidies, Copy number variants, Microarray

## Abstract

**Background:**

Noninvasive prenatal testing (NIPT) allows for screening of fetal aneuploidy and copy number variants (CNVs) from cell-free DNA (cfDNA) in maternal plasma. Professional societies have not yet embraced NIPT for fetal CNVs, citing a need for additional performance data. A clinically available genome-wide cfDNA test screens for fetal aneuploidy and CNVs larger than 7 megabases (Mb).

**Results:**

This study reviews 701 pregnancies with “high risk” indications for fetal aneuploidy which underwent both genome-wide cfDNA and prenatal microarray. For aneuploidies and CNVs considered ‘in-scope’ for the cfDNA test (CNVs ≥ 7 Mb and select microdeletions), sensitivity and specificity was 93.8% and 97.3% respectively, with positive and negative predictive values of 63.8% and 99.7% as compared to microarray. When including ‘out-of-scope’ CNVs on array as false negatives, the sensitivity of cfDNA falls to 48.3%. If only pathogenic out-of-scope CNVs are treated as false negatives, the sensitivity is 63.8%. Of the out-of-scope CNVs identified by array smaller than 7 Mb, 50% were classified as variants of uncertain significance (VUS), with an overall VUS rate in the study of 2.29%.

**Conclusions:**

While microarray provides the most robust assessment of fetal CNVs, this study suggests that genome-wide cfDNA can reliably screen for large CNVs in a high-risk cohort. Informed consent and adequate pretest counseling are essential to ensuring patients understand the benefits and limitations of all prenatal testing and screening options.

**Supplementary Information:**

The online version contains supplementary material available at 10.1186/s13039-023-00642-4.

## Background

The discovery of circulating fetal cell free DNA (cfDNA) in maternal plasma led to new prenatal screening modalities. Noninvasive prenatal testing (NIPT) methods were introduced in 2011 and have since become a widely accepted screening tool due to their enhanced performance when compared with traditional serum screening [1, 2]. In the event of an abnormal cfDNA screening result, professional society guidelines recommend confirmatory diagnostic testing via amniocentesis or chorionic villus sampling (CVS) [1].Traditional cfDNA screening is typically limited to trisomies 21, 18, 13, and may also include sex chromosome aneuploidies and a select group of microdeletions. Data suggests that approximately 80% of pregnancies with chromosome abnormalities identifiable by karyotype in a general obstetric population would be identified with traditional cfDNA screening, missing approximately 20%, or one in five abnormalities [3, 4].

The most comprehensive information about the genetic health of a fetus can be obtained by testing a sample of chorionic villi collected via chorionic villus sampling (CVS) or amniocytes collected via amniocentesis. The sample obtained from either diagnostic procedure can then be sent for karyotype and/or microarray analysis. Some professional societies support offering prenatal diagnosis with karyotype or microarray to all women, regardless of age or risk factors [1, 5] and recommend microarray analysis in cases where ultrasound anomalies are detected [6]. While these diagnostic tests provide the most comprehensive assessment of fetal chromosome aberrations, some patients will decline these options due to the risk of pregnancy loss [7, 8]. Recent evidence suggests this risk may be smaller than historically believed, and perhaps even negligible compared to controls [9].

In 2015, a cfDNA screening test became available that aimed to narrow the detection gap between “traditional” cfDNA screening and diagnostic testing by utilizing genome-wide analysis of aneuploidy and copy-number variations (CNVs) equal to or greater than 7 Mb, as well as a select group of microdeletions [10]. This genome-wide cfDNA screening test can be offered to patients as an alternative to “traditional” cfDNA screening when more information is desired, but diagnostic testing is declined. Although the 7 Mb threshold makes the genome-wide cfDNA assay more comparable to a karyotype, this study compares the clinical performance of genome-wide cfDNA screening with the diagnostic standard of microarray analysis.

## Results

### Overall sample cohort

The study cohort comprised 701 unique samples meeting the inclusion criteria, with confirmation of the presence or absence of fetal CNVs by microarray from either CVS, amniocentesis, or POC (products of conception) samples (Table 1). Concurrent diagnostic methods (singly or in combination) included karyotype (46.4% of samples), FISH (fluorescent in situ hybridization, 35.5% of samples), and qfPCR (quantitative fluorescence polymerase chain reaction, 31.4% of samples) (Table 1). The indications for testing for these 701 samples are provided in Table 2. Of the 701 samples processed for analysis, 663 were reportable (94.6%). Of the non-reportable samples, 17 (2.4%) had insufficient fetal fraction as determined by the signal-to-noise ratio algorithms, and 21 (3%) were non-reportable due to technical issues. Thus, 663 samples were available for performance calculations.

**Table 1:** Maternal demographics and diagnostic testing data

Demographic	Median	Range
Maternal Age (yr)	33.2	18.3–48.6
Gestational age (wk)	17.65	10–34.29
Maternal weight (lbs)	149.5	80–367

**Procedure**	**Percent**	**Total**
CVS	15.4	108
Amniocentesis (transplacental)	13.0	91
Amniocentesis (non-transplacental)	71.0	498
Both CVS and amniocentesis	0.4	3
Products of conception	0.1	1

**Diagnostic method(s)**	**Percent**	**Total**
Karyotype + Microarray	17.3	121
Karyotype + FISH + Microarray	28.1	197
Karyotype + qfPCR + Microarray	1.0	7
FISH + Microarray	7.4	52
qfPCR + Microarray	30.4	213
Microarray	15.8	111

CVS = chorionic villus sampling, FISH = fluorescence in situ hybridization, qfPCR = quantitative fluorescent polymerase chain reaction

**Table 2:** Indications for testing

Screening indication	N (Percent)	Percent
Maternal age > 35	142	20.3
Positive serum screening	131	18.7
Ultrasound finding(s)	208	29.7
Personal/family history of CNV or aneuploidy	12	1.7
Other	19	2.7
Multiple indications	189	27.0
Total samples	701	

### cfDNA reportable breakdown

**Fig. 1:**
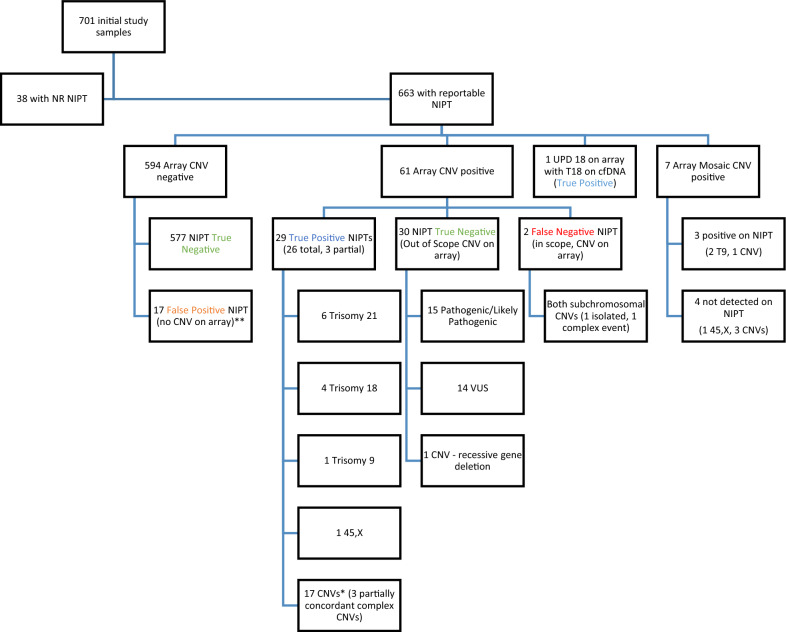
Overview of study samples and classifications/results. *Includes a case of a suspected maternal 22q deletion on cfDNA confirmed in fetus to be maternally inherited and a case of a suspected maternal duplication on chromosome 21 on cfDNA that was confirmed in the fetus; maternal testing was not performed. **Includes two cases of XXX on cfDNA that were reported as suspected maternal abnormalities with no maternal testing performed. NR = non-reportable, NIPT = noninvasive prenatal testing, CNV = copy number variant, VUS = variant of uncertain significance, UPD = uniparental disomy, 45,X = monosomy X, T9 = trisomy 9, T18 = trisomy 18

As shown in the flow chart in Fig. 1, based on the described process for categorizing microarray results before analysis, the 663 reportable samples included 594 samples with no CNVs. Another 61 samples had CNVs detected by microarray: 12 non-mosaic whole-chromosome aneuploidies and 49 with non-mosaic subchromosomal CNVs of varying sizes ranging from less than 100 kb (kilobases) to over 70 Mb. One case showed UPD18 (uniparental disomy 18) on array and as mentioned in the Methods, was treated as a true positive given the cfDNA result showing trisomy 18. Seven cases showed mosaicism on array (1 45,X [monosomy X], 2 T9 [trisomy 9], and 4 CNVs); four cases (1 45,X, 2 T9, 1 CNV) were detected by NIPT despite the mosaicism. However, these 7 samples were excluded from the performance calculations, bringing the cohort used in these calculations to 656. Detection of mosaic aneuploidies was predicted to be dependent on levels of mosaicism within the sample, with decreased detection at lower levels of mosaicism.

Of the 49 samples with non-mosaic subchromosomal CNVs, 38 cases showed isolated CNVs and 11 complex/multiple CNVs. Of the isolated CNVs, 11 were in-scope for the cfDNA assay (either > 7 Mb or involving one of the designated microdeletion regions). The remaining 27 isolated cases had out-of-scope CNVs, with 12/27 being less than 1 Mb in size and 15/27 between 1 and 7 Mb. The 11 complex cases included 8 samples with at least one CNV segment > 7 Mb on array and in-scope for the cfDNA assay. The remaining 3 cases had 2 CNVs < 500 kb and were considered out-of-scope for the cfDNA assay. Thus, a total of 30 subchromosomal CNVs were determined to be out-of-scope for the cfDNA assay. Additional file 1: Table S1 shows the details of the 49 subchromosomal CNV cases.

Furthermore, inheritance information for the CNVs was available for 22 cases: 10 were maternally-inherited, 6 were paternally-inherited, 5 were de novo, and 1 was known to be familial but parental origin was not specified on the array report. Of those with maternally-inherited CNVs, all samples demonstrated CNVs less than 7 Mb and all of them were outside the scope of genome-wide cfDNA detection, except one sample involving a 22q11.2 deletion. The assay correctly identified this deletion and suggested a likely maternal origin, conferring a 50% risk to the fetus; the deletion was confirmed by microarray in the fetus as a ‘maternally inherited 535 kb interstitial deletion of 22q11.21- > q11.21’. Of note, in the 27 isolated cases and 3 complex cases with out-of-scope CNVs < 7 Mb, 15/30 (50%) were classified as variants of uncertain significance (VUS). Overall, this results in a 2.29% VUS rate (15/656) on microarray for this study cohort.

### Sensitivity and specificity for CNVs

There were 656 samples with both microarray results and reportable cfDNA results used for calculating test performance as seen in Fig. 1. The seven cases with documented mosaicism on diagnostic testing were excluded from the primary performance calculations (Table 3).

**Table 3:** Performance of cfDNA whole and subchromosomal CNV detection based on microarray findings

	(A) Using ‘in-scope’ (*study standard*) N = 656	(B) Counting ‘out-of-scope’ CNVs as ‘false negatives’ N = 656
cfDNA Sensitivity	93.8% (95% CI 77.8—98.9%)	48.4% (95% CI 35.7—61.3%)
cfDNA Specificity	97.3% (95% CI 95.6—98.4%)	97.1% (95% CI 95.4—98.3%)
cfDNA PPV	63.8% (95% CI 48.5—76.9%)	63.8% (95% CI 48.5—76.9%)
cfDNA NPV	99.7% (95% CI 98.7–99.9%)	94.7% (95% CI 92.6—96.3%)

*(*A): Performance as measured by cfDNA with direct comparison to array outcome (study scope), treating out-of-scope CNV cases as ‘true negatives’

(B) Modification of performance if out-of-scope CNV cases are treated as ‘false negative’

*CNV* copy number variant; *PPV*  positive predictive value; *NPV* negative predictive value

Of the 594 samples negative for CNVs by microarray, genome-wide cfDNA correctly reported 577 samples negative and incorrectly reported 17 as positive (false positives). Additionally, 30 cases with normal (negative) cfDNA results, but with an out-of-scope CNV on array were treated as true negatives. This resulted in a specificity of 97.3% as shown in Table 3. Of the 32 microarray-positive, non-mosaic samples determined to fall within the scope of genome-wide cfDNA (including the 1 UPDT18/T18 case), 30 were detected on cfDNA and two samples showed no detected CNVs on cfDNA, for a sensitivity of 93.8% as shown in Table 3. The two ‘false negative’ samples were further assessed as part of the described adjudication process below to gain insight into the etiology of the discordance. The 2 × 2 contingency tables used to calculate these performance metrics are available in the (Additional file 1: Table S2).

**Fig. 2:**
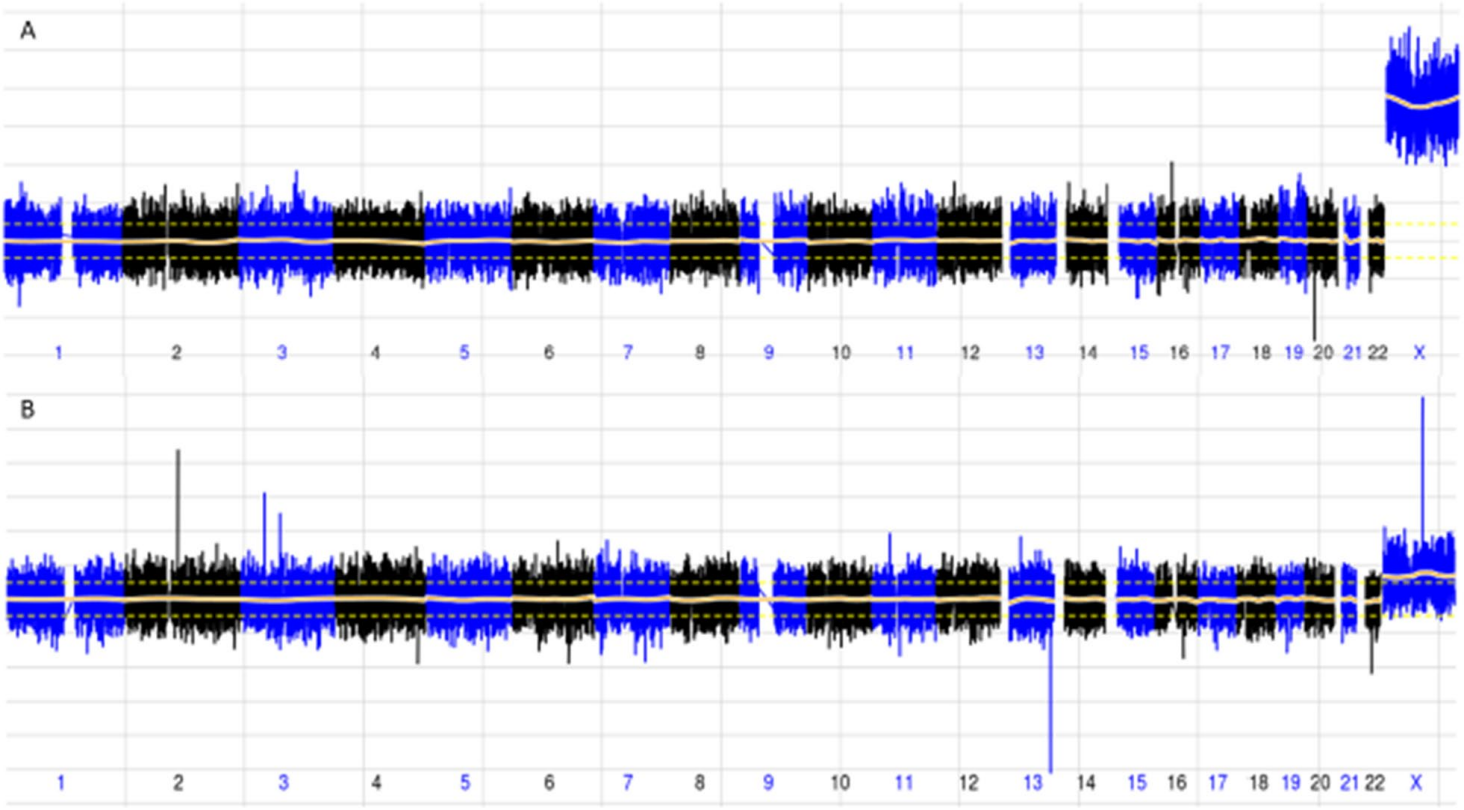
Comparison of traces for suspected maternal versus suspected fetal 47,XXX (**A**) A 50 kb trace demonstrating cfDNA sequencing data suspicious for maternal contribution, given the marked overrepresentation of X chromosome material. (**B**) A 50 kb trace from a case of confirmed fetal 47,XXX for comparison

Of the 17 ‘false positives’ (Table 4), two samples were reported as 47,XXX with high likelihood of maternal mosaicism, because there was an approximate 50% increase in chromosome X representation based on normalized sequencing data. Figure 2 shows the striking difference between a typical case of confirmed fetal 47,XXX and one of the above cases from this cohort suspicious for maternal mosaicism. No information on maternal testing or phenotype was available for these two cases. The remaining 15 samples were comprised of two samples with reported subchromosomal CNVs, one 45,X case, and 12 samples with whole chromosomal overrepresentation suggesting trisomies of chromosomes 7, 13, 14, 16, 21, or 22, and one sample with double trisomy of chromosomes 7 and 21.

**Table 4:** Details of samples with discordant array and NIPT CNV results

Sample ID	Array result	Diagnostic procedure	Fetal fraction	cfDNA reported result	uni/biplex result
45164	7p22.3p15.2(43,360–26,275,617) × 3, 7p15.2(26,275,995–26,341,643) × 1, 15q26.2q26.3(98,178,487–102,429,112) × 1	Amnio	0.125	Negative	del15q26.2q26.3
76383	15q21.2q26.3 (50,945,434–102,531,392) × 3	Amnio	0.080	Negative	Negative
45287	arr(1–22,X) × 2	CVS	0.109	45,X	45,X
40681	arr(1–22,X) × 2	Amnio	0.089	47,XXX (suspected maternal)	NA
77361	arr(1–22,X) × 2	Amnio	0.046	47,XXX (suspected maternal)	NA
76393	arr(1–22,X) × 2	Amnio	0.049	del15q11.2-q13.1	Negative
94767	arr(1–22) × 2,(XY) × 1	Amnio	0.054	del5p15	Negative
76177	arr(1–22) × 2,(XY) × 1	Amnio	0.095	T13 (mosaic)	T13 (mosaic)
93941	arr(1–22,X) × 2	Amnio	0.045	T14	T14 (mosaic)
76354	arr(1–22) × 2,(XY) × 1	Amnio	0.125	T16	T16
76178	arr(1–22,X) × 2	Amnio	0.068	T16	T16
94932	arr(1–22,X) × 2	Amnio	0.169	T16	T16
76028	arr(1–22) × 2,(XY) × 1	Amnio	0.095	T16 (mosaic)	T16 (mosaic)
54712	arr(1–22,X) × 2	Amnio	0.102	T21 (mosaic)	T21 (mosaic)
54705	arr(1–22) × 2,(XY) × 1	CVS	0.077	T22 (mosaic)	Failed re-sequencing
82511	arr(1–22,X) × 2	Amnio	0.176	T7	T7
94639	arr(1–22,X) × 2	Amnio	0.124	T7 (mosaic)	T7
94666	arr(1–22,X) × 2	Amnio	0.108	T7 (mosaic)	T7 (mosaic)
40637	arr(1–22) × 2,(XY) × 1	Amnio	0.118	T7/T21 (mosaic)	T7/T21 (mosaic)

*cfDNA* cell-free DNA, *NA* not applicable, *CVS* chorionic villus sampling, *T13* trisomy 13, *T14* trisomy 14, *T16* trisomy 16, *T21* trisomy 21, *T22* trisomy 22, *T7* trisomy 7

For the most conservative comparison of the performance of microarray to genome-wide cfDNA, performance could be calculated treating all ‘out-of-scope’ CNVs as false negatives in the performance calculations (Table 3). Given these parameters, the sensitivity of cfDNA falls to 48.3% with this conservative comparison to microarray. However, it should be noted that many cases with VUS may not result in clinical significance. As noted above, 50% (15/30) of the out-of-scope CNVs were classified as VUS. If only pathogenic/likely pathogenic CNVs were treated as false negatives, the sensitivity would be 63.8%. The details for the performance metrics and the 2 × 2 contingency tables used to calculate these performance metrics are available in the Additional File (Additional file 1: Tables S3–S5).

### Adjudication of discordant results

A total of 17 discordant cases were subjected to adjudication by uniplex (or biplex) sequencing (Table 4), in which a single sample or two samples were re-sequenced alone (or with one other sample) allowing for higher coverage. As noted above, two cfDNA samples were classified as false negatives. Array analysis of the first sample (#76383) indicated an approximately 51 Mb duplication CNV impacting 15q21.2q26.3. Uniplex sequencing of this sample showed no duplication event in this region. The second false negative sample (#45164) was determined by array to possess multiple CNVs: a 26.28 Mb terminal duplication of 7p16.2-pter, a 66 kb interstitial deletion of 7p15.2, and a 4.35 Mb terminal deletion of 15q26.2-qter. Uniplex sequencing of this sample showed no detectable 26 Mb duplication of 7p22.3p15.1 (or small deletion of 7p15.2), but did detect a 4.1 Mb deletion of 15q26.2q26.3, similar to array. With detailed review of the original cfDNA sequencing data, the 4.1 Mb deletion was flagged by the underlying algorithms of the assay, but was beyond the reportable scope of CNVs for the assay (i.e. < 7 Mb in size) and thus, not reported. In both cases, neither large duplication was seen on uniplex sampling, suggesting the possibility of a complex rescue mechanism in the placental cell lines only, resulting in absence of the CNVs in the tissues interrogated by cfDNA. This would be a biological limitation of cfDNA, rather than a limitation of the assay.

Fifteen cfDNA results classified as ‘false positive’ were also adjudicated using uniplex sequencing (Table 4). (There were two false positives excluded from adjudication, both 47,XXX, as the cfDNA sequencing data suggested maternal mosaicism as a likely etiology and resequencing would not provide further clarification). One sample (originally positive for trisomy 22) failed resequencing. In each of the 11 samples positive for one or more whole-chromosome trisomies from standard sequencing, the same trisomies were detected with similar levels of signal in the uniplexed specimens. Diagnostic outcomes were determined by amniocentesis for 10 of these 11 specimens, and the most likely explanation of disparity between array and cfDNA outcomes is confined placental mosaicism, although unrecognized co-twin demise or other biological explanations cannot be excluded. One false positive result (45,X) was also adjudicated with uniplex sequencing. Higher depth sequencing of sample #45,287 confirmed the 45,X cfDNA-based classification. While the possibility of maternal mosaic 45,X exists, the diagnostic outcome was derived from CVS so this sample is included as a false positive to maintain a conservative estimate of specificity.

Lastly, there were two samples classified as ‘false positive’ for a subchromosomal CNV and subjected to uniplex sequencing (Table 4). Both samples showed no evidence of the reported CNVs with high depth sequencing, indicating these CNVs events were indeed false positives. This adjudication process brings the number of false positives from 17 to 4. The adjudicated technical specificity of genome-wide cfDNA is estimated to be 99.3% (95% confidence interval: 98.2%-99.8%). To calculate an adjudicated sensitivity, if the uniplex results were treated as ‘truth’, the sensitivity of in-scope events would be > 99%. However, in clinical practice, cfDNA screening must also take into account not only assay limitations, but also the very real biological limitations that can lead to these discordant results. This adjudication was helpful in discerning those differences and may be useful in future practice for counseling and improving future iterations of cfDNA assays; the adjudication process was not intended to supplant the original performance calculations. The details of the adjudicated performance metrics and the 2 × 2 contingency table used for these calculations can be found in the Additional File (Additional file 1: Tables S6 and S7).

### CNV mapping precision by genome-wide cfDNA

Currently, microarray analysis for CNV detection provides precise mapping of genomic coordinates defining the start and end of a CNV. This precision enables increased confidence in genetic counseling of patients to communicate with clarity the likelihood of a CNV involving clinically significant genes, as opposed to a region with genes of unknown or benign significance.

**Fig. 3:**
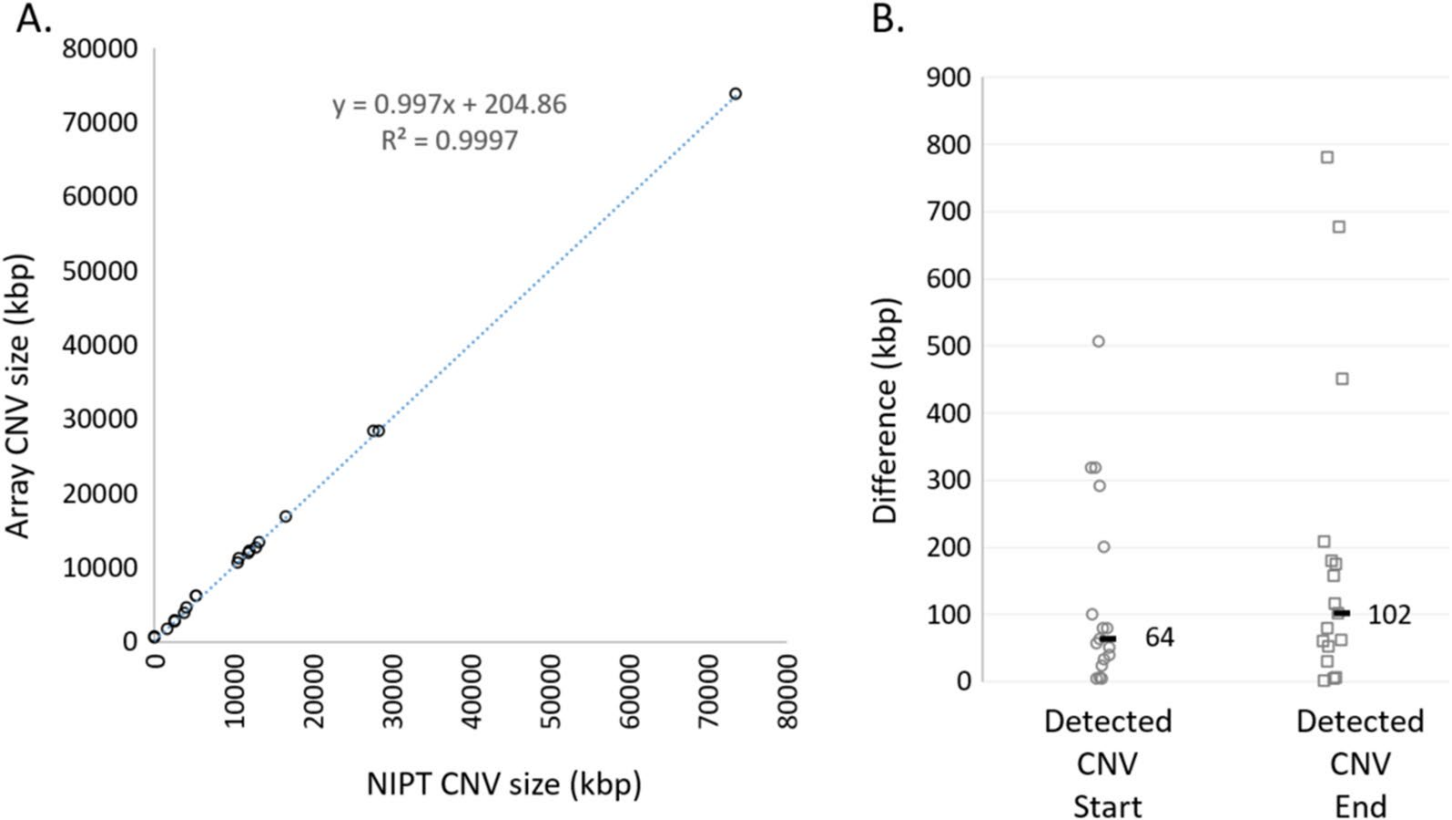
Comparison of genome wide subchromosomal CNV sizes and breakpoints between array and cfDNA reported findings. **A** Size comparison for cfDNA (x-axis) and microarray (y-axis) detected and reported CNVs. **B** Precision of the start and end genomic coordinates of CNVs detected by cfDNA relative to the array defined start and end CNV genomic coordinates. NIPT = noninvasive prenatal testing, bp = base pairs, CNV = copy number variant, kbp = kilo base pairs

To determine the precision of CNV calling by genome-wide cfDNA, the 17 cfDNA cases with positive results for a subchromosomal CNV and confirmed microarray outcomes were compiled. CNV sizes and start/end coordinates of the event, as predicted by cfDNA, were compared to the same metrics from the diagnostic results. Within these 17 samples, 19 CNVs were detected by cfDNA. Comparison of CNV size and precision of the start and end coordinates are shown in Fig. 3. Using linear regression, overall CNV sizes were highly concordant and consistent between array and cfDNA findings with an r-square > 0.99, with a slope of nearly 1.0 (Fig. 3A). Precision of mapping the start and end coordinates is limited in genome-wide cfDNA due to the use of ‘bins’ comprised of 50 kb units of genomic DNA, at least in the current method [10]. As such the highest resolution with cfDNA is ± 50 kb. In the current study, the median difference of CNV starting coordinates was 67 kb and 102 kb for CNV end coordinates (Fig. 3B), highlighting the precision that can be achieved with cfDNA analysis for CNVs.

## Discussion

### Test performance

This study provides a direct comparison of CNV detection between genome-wide low coverage whole genome sequencing of maternal plasma cfDNA versus microarray analysis of amniocytes, chorionic villi, or products of conception. By the very nature of sample types and methods, such an assessment is difficult and is the primary limitation of the comparison attempted here. The analyte in cfDNA (from the trophoblast of the placenta) can be interrogated by direct CVS, but differentiates very early in embryonic development from the cells interrogated by amniocentesis. While amniocytes are most representative of fetal DNA and thus most useful for determining fetal status, CVS may be a better comparator for assessing cfDNA performance, since both are analyzing placental DNA. Amniocentesis was the predominant diagnostic test in this cohort, thus, discordant results cannot truly exclude the presence of the event detected by cfDNA in the placenta.

As seen in Table 3, based on the study parameters, genome-wide cfDNA shows a high sensitivity, specificity, PPV, and NPV for chromosome abnormalities within the scope of the assay, as compared to microarray. The test performance in this study is similar to previous studies on the same assay [10–12], as well as studies utilizing different genome-wide cfDNA assays [13–17], although those studies included both karyotype and microarray in performance assessment. The PPVs for CNVs alone in the studies utilizing the same assay are ~ 72%-74% for CNVs at least 7 Mb, with much larger cohorts that were still predominantly high-risk patients; however, those were both retrospective studies with different protocols for collecting and comparing the diagnostic testing outcomes, which may have biased the test performance [11, 12]. Other studies using different genome-wide assays explored performance in both high-risk and general obstetric populations. Fiorentino et al. (2017) found in a general obstetric population, comprised of both high-risk and average-risk patients, that genome-wide cfDNA had a sensitivity of 100% for rare trisomies, segmental imbalances (CNVs), and common aneuploidies, with a specificity of 99.94% for rare trisomies and 99.96% for segmental imbalances [13]. The TRIDENT studies in the Netherlands have explored performance of genome-wide NIPT in both high-risk and general obstetric populations and have also explored the origin and clinical impact of the genome-wide findings (‘additional findings’) beyond the common trisomies [14–17]. TRIDENT-2, which explored the performance of genome-wide NIPT as a first-tier screen offered to all pregnant patients found that ‘additional findings’ were present in 0.36% of patients[17]. PPVs were lower for rare trisomies (~ 4–6%) but found that even ‘false positive’ cases may still have clinical impact due to confined placental mosaicism [16–18]. Fetal segmental imbalances (CNVs) had PPVs of ~ 32%-47% in the general population and were often associated with severe phenotypes [16–18]. The data across all of these studies, including the current study, suggests that the assay is largely successful in detecting the expected chromosome abnormalities and that positive results on cfDNA are often true positives, yet performance in the general obstetric population is lower than in a high-risk cohort, as is to be expected. Yet, it is important to note that even when limiting the comparison to ‘in-scope’ abnormalities for the cfDNA test, there were still both false positive and false negative cases. This underscores the importance of diagnostic testing with microarray as the gold standard for detection of fetal aneuploidy and copy number variants in pregnancy.

For maternally inherited CNVs on cfDNA, the fraction of impacted maternal DNA in the cfDNA sample generates a very strong signal of increased or decreased chromosomal representation and precludes the ability to determine the presence of the inherited CNV in the fetal genome. Detection of a maternal CNV by cfDNA would confer a subsequent risk of 50% for the fetus to inherit the CNV and copy number assessment of the rest of the genome is unhindered. In this cohort, 4 cases [2 XXX (unconfirmed), 1 22q del (confirmed), and a 9.81 Mb CNV on chromosome 21 (confirmed in fetus, no maternal testing)] were suspected to be maternal events based on the strength of the signal in the cfDNA sequencing data and the cfDNA report suggested a likely maternal origin for the finding. In the case of the chromosome 21 CNV, the patient had a previous NIPT at an outside lab suggestive of trisomy 21. The genome-wide cfDNA was able to be more specific, noting a large CNV (instead of aneuploidy) and the likely maternal origin. These specific details could be beneficial in guiding appropriate follow-up testing.

Along those lines, of the out-of-scope CNVs < 7 Mb, 50% (15/30) were classified as variants of uncertain significance (VUS), underscoring an important counseling point for patients considering microarray. The chosen 7 Mb threshold for the cfDNA assay was selected in part to try and minimize situations in which a VUS would be reported by a screening test. In this study cohort, none of the VUS detected by microarray were larger than 7 Mb. It is important to note that the number of VUS detected in this study may be due to the criteria used for reporting at the array laboratories. Currently, there is no consensus or standard for a reporting cutoff to identify a VUS in the prenatal setting, which is an important consideration when discussing the clinical implications of this testing [19].

### CNV size and location mapping

Analysis of CNV sizes detected by both array and cfDNA were highly concordant (Fig. 3) and median start/end genomic coordinates of CNVs were typically within 60–120 kb of each other. A cfDNA assay which provides information about size and location of CNVs may aid in counseling regarding the clinical impact of a given CNV based on the presence or absence of known disease-associated genes in the CNV region. However, bin size on cfDNA is the limiting factor in breakpoint precision in that breakpoint estimates will only be as good as the minimum size of a bin. Microarray is able to clarify the inclusion or exclusion of specific genes within the CNV at the breakpoints estimated by cfDNA. Future directions of cfDNA analysis could potentially increase precision of CNV coordinates and, at the same time, greater sensitivity to detect smaller CNVs by use of higher sequencing depths. However, the practical utility of a higher resolution cfDNA assay for smaller CNV detection remains to be determined. One area for further study could be to explore whether microarray identifies clinically significant genes that are not included in the CNV breakpoints estimated by cfDNA.

### Limitations

One of the main limitations of the study is the sampling bias. Patients included in the study cohort were at increased risk for aneuploidy with a variety of indications and the study cohort is entirely comprised of patients already scheduled to undergo a diagnostic procedure. Patients who have a positive FISH, qfPCR, or karyotype may not proceed to microarray, so this cohort may reflect an underrepresentation of the core aneuploidies. Given these limitations, this study cohort is likely not representative of the general pregnancy population, although it is reasonably representative of the patient population that pursues diagnostic testing. Finally, the microarrays were performed as part of a patient’s clinical care at a number of institutions; thus, the type of array platforms utilized and subsequent interpretation were not controlled for in the study. Labs may utilize slightly different reporting rules and thresholds, which could influence the array findings in the study.

Multiple biological mechanisms can result in discordant cfDNA results, including co-twin demise, mosaicism, and maternal abnormalities, among others. For this study, only the number of fetuses at the time of sampling (singletons in this cohort) was available as indicated by the ordering provider, and no information on potential co-twin demise was known. Thus, co-twin demise cannot be excluded as an explanation for discordant results, particularly for false positives. Furthermore, mosaicism, especially confined placental mosaicism, is a confounding factor when comparing cfDNA results to those of an amniocentesis. Mosaicism can lead to discordant results presenting as both ‘false positives’ due to confined placental mosaicism (CPM) and ‘false negatives’ when a chromosome abnormality is confined to the mesenchymal layer of the placenta or to the fetus. Even mosaicism present in trophoblast which is interrogated by a cfDNA assay may evade detection depending on the degree of mosaicism and fetal fraction of the specimen. Because this study did not include obstetric or neonatal outcomes and follow-up, an assessment of the clinical relevance of discordant results was not possible. Literature suggests that CPM may present an increased risk for adverse pregnancy outcomes depending on the timing and origin of the nondisjunction and the chromosome involved, with particular focus on trisomy 16 [20–23].

Along these lines, one case showed T18 on cfDNA and UPD18(mat) on microarray on amniotic fluid. As a major mechanism of UPD is trisomic rescue, the combination of T18 on cfDNA and UPD18 on amniotic fluid represents a typical example of trisomic rescue in prenatal diagnosis [24]. As described above, T18 in the placental cell lines may confer a risk for adverse pregnancy outcomes, while UPD18 in the fetus may or may not have clinical significance [15, 20–24]. Chromosome 18 is not associated with any known imprinting disorders, although there is residual risk for an autosomal recessive disorder [24]. UPD may also be associated with an increased risk for adverse pregnancy outcomes [23]. If this case were treated as a false positive, the test performance remains similar, with a sensitivity of 93.5%, specificity of 97.1%, a PPV of 61.7%, and an NPV of 99.7%, as detailed in the Additional File (Additional file 1: Tables S8 and S9).

Of the CNV cohort, 30 array-positive cases were determined to fall outside of the scope of the current genome-wide cfDNA assay, highlighting the sensitivity of microarray analysis to detect small microdeletions as compared to current cfDNA assays. However, 50% of the out-of-scope events were classified as VUS with most, if not all, of these below the resolution of a karyotype. While the overall VUS rate for prenatal microarray is dependent on the platform and laboratory reporting criteria, the VUS rate among the ‘out-of-scope’ events in this study may be helpful for patient decision making, both regarding cfDNA and diagnostic testing, and when choosing between karyotype and microarray.

Theoretically, the ‘undetected’ maternal CNVs < 7 Mb would have precluded fetal analysis regardless of cfDNA detection due to the strength of the maternal signal, similarly to the detected 22q deletion. But if detected by cfDNA, these findings could alert to a 50% risk for inheritance and prompt further evaluation of the fetus during the pregnancy or after birth. In some cases, information about possible maternal chromosome abnormalities may be considered beneficial to the pregnant patient, while in others, the unanticipated information may be considered less desirable. Pretest counseling is critical and may help prepare patients undergoing cfDNA for the possibility of incidental identification of a maternal chromosome abnormality. Expanding the scope of cfDNA will by definition find more maternal abnormalities and the ethical and clinical impact should be considered.

The non-reportable rate seen in this study cohort is higher than that of the “traditional” cfDNA assay for common aneuploidies of 0.9% [25, 26] and is slightly higher than seen in a large clinical cohort of over 55,000 samples using the same assay [11]. However, genome-wide CNV detection requires more robust sequencing data with less noise and artifact as compared to the traditional aneuploidies. As such, a higher signal to noise ratio (SNR) is required for samples to be reportable by the genome-wide assay as compared to the SNR required for the traditional cfDNA assay. Increasing the number of sequencing reads per sample can help overcome this limitation to an extent, but with the trade-off of increasing the cost per sample.

## Conclusions

Undoubtedly, microarray via amniocentesis provides a more robust assessment of the fetal chromosome complement compared to cfDNA screening, and is deservedly the standard for prenatal detection of fetal chromosome conditions. The current cfDNA assay was not intended to meet the level of clinical information provided by microarray, as evidenced by the ~ 48% detection rate of cfDNA when ‘out-of-scope’ CNVs were considered. However, some portion of women will continue to decline diagnostic testing or face logistical or financial challenges in accessing a diagnostic procedure. For those patients, a screening option which provides more clinically relevant screening information may be advantageous. The current study shows that the genome-wide cfDNA assay performs as expected, finding most chromosome abnormalities within the scope of the test. However, diagnostic testing is still needed to confirm screen-positive results, or to provide the level of detail available by microarray. Future studies could further explore the clinical utility of using a genome-wide cfDNA assay to screen for events smaller than 7 Mb and the impact of a dropping the size threshold on test performance.

## Methods

### Study cohort

Subjects included pregnant women at increased risk for fetal aneuploidy based on advanced maternal age (≥ 35 years), a positive serum screen, an abnormal ultrasound finding, and/or a history of aneuploidy who were scheduled to undergo amniocentesis and/or CVS to determine fetal chromosomal status. Table 1 summarizes the maternal demographics and diagnostic methods, while Table 2 reviews the testing indications. Maternal whole blood samples were collected into 10 mL EDTA anti-coagulated whole blood tubes containing a cell stabilizer (Streck cfDNA BCT, Omaha, NE).

Samples from the clinical trial databases were reviewed for possible inclusion in the study. To be included, fetal chromosomal status as determined by microarray analysis on amniotic fluid (AF), chorionic villi (CV), or products of conception was required. Samples with low banked plasma volumes, blood-to-plasma processing times above 7 days, or with unavailable/failed array results were also excluded. Ultimately, 701 samples were eligible for inclusion in the study analysis.

### Sample processing

Maternal blood samples were collected at IRB (institutional review board) approved clinical sites, shipped to Sequenom Inc. (San Diego, CA) at ambient temperature and processed to collect the plasma fraction of each sample as previously described [27]. Plasma aliquots were stored frozen at − 80 °C. Plasma samples included in the study required volumes ≥ 3 mL to allow for analysis of a second aliquot if needed and must have been processed to plasma within 7 days of blood collection.

All plasma analyses and result reviews were completed in a CAP/CLIA accredited laboratory following the processes in place for commercial genome-wide cfDNA samples. At the time of the study, frozen plasma aliquots were retrieved from storage, thawed and centrifuged; the supernatant was transferred to processing tubes. Samples were extracted, processed, and analyzed for whole chromosome aneuploidies and subchromosomal CNVs ≥ 7 Mb as well as select microdeletions < 7 Mb associated with 1p36 deletion, Wolf–Hirschhorn (4p16.3), Cri-du-chat (5p15.2), Langer–Giedion (8q23.2q24.1), Jacobsen (11q24.1), Prader-Willi/Angelman (15q11q13), and DiGeorge (22q11.2) syndromes as previously described [10, 27–29], with fetal fractions determined as previously described [30]. Samples with insufficient fetal fraction as determined relative to sequencing noise were considered non-reportable (QNS), while samples which failed to meet other laboratory quality metrics were considered non-reportable for technical reasons [31]. One of the challenges of cfDNA CNV detection is that typically only about 5–15% of the DNA of interest is ‘affected’ with the CNV, while microarray has no such limitations and is able to detect CNVs as small as 25–50 kb depending on local probe density within a particular genomic region. A 7 Mb threshold for subchromosomal events is currently utilized by the cfDNA laboratory in order to maintain a high sensitivity and specificity for the screening assay while also focusing on information that is likely to be clinically significant based solely on the size of the event.

### Data review

Results from sequencing and analysis algorithms were reviewed in a blinded manner by Clinical Laboratory Directors following standard laboratory practices as defined for reporting the results obtained through the genome-wide cfDNA assay.

The microarray platforms utilized and the interpretation of any microarray findings were not controlled for in the current study, as these were part of the clinical pregnancy management by independent physicians and laboratories. However, ~ 95% of arrays were performed using the ThermoFisher® [Affymetrix® during the study period] CytoScan® HD array [ThermoFisher ® and CytoScan® are Registered Trademarks of ThermoFisher, Inc.], which uses approximately 2.695 million markers across the genome. There are approximately 743,000 single nucleotide polymorphic probes (SNPs) and 1,953,000 structural non-polymorphic probes. A detailed summary of the various array platforms in the study as extracted from the clinical reports may be found in Additional File 1 (Additional file 1: Table S10). Samples may have also been analyzed by karyotype, fluorescence in situ hybridization (FISH), or quantitative fluorescence PCR (qfPCR). Supplementary karyotype, FISH, and/or qfPCR outcomes were considered in context to clarify samples with balanced structural rearrangements and no copy number gain or loss, however, microarray was the focus of the test comparison.

Genome-wide cfDNA screening results were compared to the microarray result documented for each sample in the clinical databases following a defined unblinding plan as preapproved by both the internal and external authors. The genome-wide cfDNA algorithms used in the study analyze for whole chromosome aneuploidies, genome-wide CNVs ≥ 7 Mb, as well as specific deletions < 7 Mb associated with microdeletion syndromes located at 1p36, 4p16.3, 5p15.2, 8q23.2q24.1, 11q24.1, 15q11q13, and 22q11.2 [10]. As microarray reliably detects CNVs as small at 25–50 kb as well as regions of homozygosity (ROH) when SNP probes are used, microarray is expected to outperform cfDNA screening. Prior to analysis of cfDNA from plasma samples, de-identified microarray results were reviewed by a subset of co-authors and the samples were stratified into three groups: absence of any CNV (this group includes samples with ROH or balanced structural variants); presence of a whole chromosome aneuploidy; or presence of a subchromosomal CNV. Those with a subchromosomal CNV were further scrutinized to determine if the events would fall within or outside the scope of the genome-wide cfDNA assay, with subchromosomal CNVs < 7 Mb outside of the select microdeletion regions above considered outside the scope of the genome-wide cfDNA methods.

For the purposes of calculating test performance, samples were considered a ‘true positive’ if a CNV detected by the cfDNA assay was confirmed with diagnostic testing. One sample showed UPD of chromosome 18 on array and the cfDNA showed trisomy 18 and was treated as a true positive, as the likely explanation is a trisomic rescue in the fetus. Samples were considered a ‘false positive’ if the CNV identified by cfDNA screening was not detected by diagnostic testing. Samples were considered a ‘true negative’ if the genome-wide cfDNA result was negative and was concordant with the microarray result. If the microarray finding was determined to be ‘out-of-scope’ for the cfDNA assay (i.e. below the detection resolution of 7 Mb or ROH) and was otherwise negative, these were considered a ‘negative: out-of-scope’. A ‘false negative’ was assigned when an ‘in-scope” CNV detected by microarray was not identified by the cfDNA assay. Only three cases had both CVS and amniocentesis testing during the same pregnancy, and in only one of those cases was there a discordance between diagnostic tests (mosaic UPD event on CVS and normal amniocentesis).

A two-by-two contingency table was used to calculate sensitivity, specificity, positive predictive value (PPV), and negative predictive value (NPV) using VassarStats [32].

Discordant results were examined with additional scrutiny to determine if the cfDNA findings were reproducible at a higher depth of sequencing (as previously described by Lefkowitz et al. [10]) and provided insight into a potential etiology for the discordance. Discordant specimens were subjected to uniplex sequencing, allowing for over 200 million sequencing reads dedicated solely to the specimen in question, significantly improving the quality of signal-to-noise ratio in these cases. While uniplex sequencing of samples has significant advantages in test performance, it is typically cost-prohibitive in a clinical setting.

## Supplementary Information

**Additional file1**. Contains **Table S1** which details the 49 subchromosomal CNV cases included in the performance calculations, **Tables S3–S9** which contain details of the various performance calculations, and **Table S10** which contains the details regarding the various array platforms as extracted from the laboratory report

## Data Availability

All relevant data generated or analyzed during this study are included in this published article [and its supplementary information files].

## Abbreviations

cfDNA: Cell-free DNA
NIPT: Non-invasive prenatal testing
CNV: Copy number variant
Mb: Megabase
VUS: Variant of uncertain significance
CVS: Chorionic villus sampling
POC: Products of conception
FISH: Fluorescent in situ hybridization
qfPCR: Quantitative fluorescence polymerase chain reaction
Yr: Year
Wk: Week
Lbs: Pounds
45,X: Monosomy X
UPD: Uniparental disomy
NR: Non-reportable
T9: Trisomy 9
T18: Trisomy 18
PPV: Positive predictive value
NPV: Negative predictive value
Bp: Base pairs
Kbp: Kilo base pairs
CPM: Confined placental mosaicism
SNR: Signal to noise ratio
mL: Milliliter
EDTA: Etheylenediaminetetraacetic acid
AF: Amniotic fluid
CV: Chorionic villi
IRB: Institutional Review Board
CAP/CLIA: College of American Pathologists/Clinical Laboratory Improvement Amendments
T13: Trisomy 13
T14: Trisomy 14
T16: Trisomy 16
T21: Trisomy 21
T22: Trisomy 22
T7: Trisomy 7
