# Supplementary material for: Laboratory performance of genome-wide cfDNA for copy number variants as compared to prenatal microarray
Source: Mol Cytogenet. 2023 Jun 10;16:10. doi: 10.1186/s13039-023-00642-4 (PMC10257834; doi:10.1186/s13039-023-00642-4)
Supplement: Supplementary file 1 — Additional file1. Contains Table S1 which details the 49 subchromosomal CNV cases included in the performance calculations, Tables S3–S9 which contain details of the various performance calculations, and Table S10 which contains the details regarding the various array platforms as extracted from the laboratory report [file 13039_2023_642_MOESM1_ESM.pdf]

Supporting Information for Soster et al, "Laboratory performance of genome-wide cfDNA for copy number variants as compared to prenatal microarray"

Supplemental Table S1: Details of the 49 subchromosomal CNV cases included in the performance metric calculations

Sample ID	Array Result	Diagnostic Procedure	cfDNA reported result	For subchromosomal CNVs		
				CNV size	*CNV inheritance*	*CNV Significance*
45164	26.28 Mb duplication of 7pter-p15.2; 66 kb deletion of 7p15.2-p15.2; 4.35 Mb deletion of 15q26.2-qter	Amnio	Negative	26.28 Mb; 66 kb; 4.35 Mb	Unknown	Pathogenic
53062	12.1 Mb duplication of 11p15.55-p15.3 and 364 kb deletion of 11q25	CVS	~12 Mb duplication of 11p15.5-p15.3	12.1 Mb; 364 Kb	Unknown	Pathogenic
53477	10.4 Mb deletion of 3p26.3-p25.3	Amnio	~10.5 Mb deletion of 3p26.3-p25.3	10.4 Mb	De novo	Pathogenic
53825	655 kb duplication of 16p11.2	Amnio	Negative	655 kb	Maternal	VUS
54723	1.4 Mb deletion of 17q12	Amnio	Negative	1.4 Mb	Maternal	Pathogenic
55463	838 kb duplication of 12q4.32-q24.33	Amnio	Negative	838 kb	Unknown	VUS
76029	2.88 Mb deletion of 22q11.21	Amnio	2.75 Mb deletion of 22q11.21	2.88 Mb	Unknown	Pathogenic

76040	2.55 Mb deletion of 22q11.21	Amnio	2.75 Mb deletion of 22q11.21	2.55 Mb	Unknown	Pathogenic
76062	9.81 Mb duplication of 21q21.1-q21.3	Amnio	9.75 Mb duplication of 21q21.1-q21.3	9.81 Mb	Unknown (NIPT suggests likely maternal)	Pathogenic
76081	655 kb deletion of 17q21.31	Amnio	Negative	655 kb	De novo	Pathogenic
76095	146 kb duplication of Xp11.4	Amnio	Negative	146 kb	Maternal	VUS
76097	6.06 Mb deletion of 15q11.2-q13.1	Amnio	5.30 Mb deletion of 15q11.2-q13.1	6.06 Mb	Unknown	Pathogenic
76101	2.35 Mb deletion of 15q25.2	Amnio	Negative	2.35 Mb	Unknown	Pathogenic
76182	11.78 Mb deletion of 4pter-p16.1	Amnio	10.90 Mb deletion of 4p16.3-p16.1	11.78 Mb	Unknown	Pathogenic
76190	1.66 Mb duplication of 22q11.21	Amnio	Negative	1.66 Mb	Paternal	Pathogenic
76306	1.62 Mb deletion of 16p13.11	Amnio	Negative	1.62 Mb	Paternal	Pathogenic
76349	84 kb deletion of 2p16.3	Amnio	Negative	84 kb	Maternal	Pathogenic
76351	86 kb deletion of 2q13	Amnio	Negative	86 kb	De novo	Recessive gene deletion*
76383	51.6 Mb duplication of 15q21.1-qter	Amnio	Negative	51.6 Mb	Unknown	Pathogenic
76437	4.35 Mb duplication of 1q31.1	Amnio	Negative	4.35 Mb	Maternal	VUS

77031	48 kb deletion of 12p12.1	Amnio	Negative	48 kb	Maternal	VUS
77199	13.4 Mb duplication of 7p21.3-p22.3; 3.7 Mb deletion of 9p24.2-p24.3	Amnio	13.25 Mb duplication of 7p21.3-p22.3	13.4 Mb; 3.7 Mb	Unknown	Pathogenic
77269	2.58 Mb duplication of 17p12	Amnio	Negative	2.58 Mb	De novo	Pathogenic
77318	1.50 Mb deletion of chromosome 7q11.23	Amnio	Negative	1.50 Mb	Unknown	Pathogenic
77334	323 kb duplication of 7q36.3 and another 184 kb duplication of 7q36.3	Amnio	Negative	323 kb, 184 kb	Unknown	Likely pathogenic
79006	6.16 Mb deletion of 15q11.2-q13.1	CVS	5.30 Mb deletion of 15q11.2-q13.1	6.16 Mb	Unknown	Pathogenic
79011	28.35 Mb deletion of 5pter-p14.1; 1.68 Mb duplication of 21q22.3-qter	CVS	28.35 Mb deletion of 5p15.33-p14.1	28.35 Mb; 1.68 Mb	Paternal (translocation)	Pathogenic
79024	Large non-contiguous complex duplication in distal arm of chromosome 16 of approximately 15Mb	CVS	9.15 Mb duplication of 16p13.2-p12.3	~15 Mb	Unknown	Pathogenic
82328	270 kb deletion of 8q23.3; 262 kb deletion of 12q21.1	CVS	Negative	270 kb; 262 kb	Familial (previous child with both deletions)	VUS

82465	11.9 Mb duplication of 2q32.1-q33.1	Amnio	11.95 Mb duplication of 2q32.1-q33.1	11.9 Mb	Paternal	Pathogenic
82483	847 kb deletion of 13q32.3	Amnio	Negative	847 kb	Unknown	Pathogenic
82514	1.10 Mb deletion of 8q24.1-q24.22	Amnio	Negative	1.10 Mb	Unknown	Pathogenic
82517	2.13 Mb deletion of 5q35.2-q35.3	Amnio	Negative	2.13 Mb	Unknown	Pathogenic
89685	1.7 Mb duplication of 1q41	Amnio	Negative	1.7 Mb	Unknown	VUS
89739	862 kb deletion of 2q13	Amnio	Negative	862 kb	Unknown	VUS
89749	260 kb deletion of 11q22.3	Amnio	Negative	260 kb	Unknown	VUS
89798	28.2 Mb deletion of 6q13.1-q21; 560 kb deletion of 16p11.2	Amnio	27.85 Mb deletion of 6q14.1-q21	28.2 Mb; 560 kb	Unknown	Pathogenic
93646	2.34 Mb duplication of 5q34	Amnio	Negative	2.34 Mb	Unknown	VUS
93680	2.68 Mb duplication of 16p13.11-p12.3	Amnio	Negative	2.68 Mb	Unknown	Pathogenic
93684	535 kb deletion of 22q11.21 (proximal to common deletion interval)	Amnio	0.5 Mb deletion of 22q11.2 (likely maternal origin)	535 kb	Maternal	VUS
93937	1.38 Mb deletion of 17p12	Amnio	Negative	1.38 Mb	Paternal	Pathogenic
94647	1.58 Mb deletion of 16q22.1-q22.2	Amnio	Negative	1.58 Mb	Unknown	Pathogenic

94654	73.8 Mb duplication of 3q21.2-q29; 4.5 Mb deletion from 15q26.2-q26.3	Amnio	73.60 Mb duplication of 3q21.2-q29	73.8 Mb; 4.5 Mb	Unknown	Pathogenic
94735	323 kb duplication of 7q36.3 and another 177 kb duplication of 7q36.3	CVS	Negative	323 kb; 177 kb	Maternal	Likely pathogenic
94843	16.81 Mb deletion of 10p15.2-p13	Amnio	16.60 Mb deletion of 10p15.3-p13	16.81 Mb	De novo	Pathogenic
94875	12.5 Mb deletion of 4q34.3-qter; 40.0 Mb duplication of 12q22-qter	Amnio	12.80 Mb deletion of 4q34.3-q35.2	12.5 Mb; 40.0 Mb	Unknown	Pathogenic
94891	469 kb deletion of 11p15.1	CVS	Negative	469 kb	Unknown	VUS
94911	314 kb triplication of Xq28	Amnio	Negative	314 kb	Paternal	VUS
94929	3.18 Mb duplication of 4q22.1-q22.2	Amnio	Negative	3.18 Mb	Maternal	VUS

**Counted with VUS category, as clinical significance is unclear without additional testing and may not have any impact on this pregnancy.*

cfDNA = cell-free DNA, CNV = copy number variant, Mb = megabase, kb = kilobase, VUS = variant of uncertain significance, CVS = chorionic villus sampling

Supplemental Table S2: 2x2 Contingency Table used to calculate the performance metrics for the assay as documented in Table 3, Column A – 'Study Standard'

	**Condition**		**Totals**
	**Absent**	**Present**	
**Test Positive**	17	30	47
**Test Negative**	607	2	609
**Totals**	624	32	656

Supplemental Table S3: 2x2 Contingency Table used to calculate the performance metrics for the assay as documented in Table 3, Column B – 'Out-of-scope as false negatives'

	Condition		Totals
	Absent	Present	
**Test Positive**	17	30	47
**Test Negative**	577	32	609
**Totals**	594	62	656

Supplemental Table S4: Performance metrics if only pathogenic/likely pathogenic CNVs are treated as false negatives

**Sensitivity**	63.8%	(95% CI: 48.5%-76.9%)
**Specificity**	97.2%	(95% CI: 95.5%-98.3%)
**PPV**	63.8%	(95% CI: 48.5%-76.9%)
**NPV**	97.2%	(95% CI: 95.5%-98.3%)

Supplemental Table S5: 2x2 Contingency Table used for Table S4

	Condition		Totals
	Absent	Present	
**Test Positive**	17	30	47
**Test Negative**	592	17	609
**Totals**	609	47	656

Supplemental Table S6: Adjudicated test performance referenced in the manuscript

**Sensitivity**	>99.9%	(95% CI: 89.8% - >99.9%)
**Specificity**	99.03%	(95% CI: 98.2%-99.8%)
**PPV**	91.5%	(95% CI: 78.7%-97.2%)
**NPV**	>99.9%	(95% CI: 99.2% - >99.9%)

Supplemental Table S7: 2x2 Contingency Table used for Table S6

	Condition		Totals
	Absent	Present	
**Test Positive**	4	43	47
**Test Negative**	609	0	609
**Totals**	613	43	656

Supplemental Table S8: Performance metrics calculated while treating the T18/UPD18 case as a false positive.

**Sensitivity**	93.5%	(95% CI: 77.2%-98.9%)
**Specificity**	97.1%	(95% CI: 95.4%-98.2%)
**PPV**	61.7%	(95% CI: 46.4%-75.1%)
**NPV**	99.7%	(95% CI: 98.7% - >99.9%)

Supplemental Table S9: 2x2 Contingency Table used for Table S8

	Condition		Totals
	Absent	Present	
**Test Positive**	18	29	47
**Test Negative**	607	2	609
**Totals**	625	31	656

Supplemental Table S10: Array platform details, as extracted from the laboratory reports. (In a small number of cases (n=9), the reporting criteria or assay details were not legible or were not available on the reports in the clinical trial databases, and are listed as UNK, for unknown)

Lab Code Name	Platform	Probes/Targets	Build	Additional details on reporting criteria per report
Lab A<br><br>N = 256	Affymetrix Cytoscan HD	2.695 Million Targets (SNP + Copy Number)	GRCh37/hg19 assembly	Gains >2 Mb and losses >1 Mb included at least one OMIM annotated gene; Gains/losses >25-50 kb within clinically significant genes/regions

Lab B<br><br>N = 310	Affymetrix Cytoscan HD	2.695 Million Targets (SNP + Copy Number)	GRCh37/hg19 assembly	Gains >1 Mb and losses >500 kb
Lab C<br><br>N = 1	Illumina CytoSNP 850K	850K SNP markers	GRCh37/hg19 assembly	Gains >200 kb and losses >100 kb; Genome-wide resolution of 18 kb, and 10 kb in the targeted disease regions (available upon request)
Lab D<br><br>N = 55	Affymetrix Cytoscan HD	2.695 Million Targets (SNP + Copy Number)	GRCh37/hg19 assembly	Gains >2 Mb and losses >1 Mb; Smaller CNVs 25-50 kb reported only if established clinical significance
Lab E<br><br>N = 20	Affymetrix Cytoscan HD	2.695 Million Targets (SNP + Copy Number)	GRCh37/hg19 assembly	Gains >500 kb and losses >250 kb; Resolution of 25-50 kb throughout the genome
Lab F<br><br>N = 1	Illumina CytoSNP-12b	~300K SNP markers "targeting regions known to be important for cytogenetic analysis" https://www.illumina.com/products/by-type/clinical-research-products/human-cytosnp-12.html	Hg18 assembly	Gains or losses >5 Mb; One of a list of known del/dup syndromes*; Terminal gains or losses < 5 Mb but > 1 Mb 'will be reviewed and reported based on clinical relevance'; Detects UPD due to heterodisomy; UPD due to isodisomy will be reported as monosomy; two copies of Y chromosome will be reported as a single Y
Lab G<br><br>N = 7	Illumina CytoSNP 850K	>845K SNP markers	GRCh37/hg19 assembly	CNVs evaluated for changes involving at least 16 probes

Lab G – Targeted<br>N = 10	Illumina CytoSNP 850K	>845K SNP markers	GRCh37/hg19 assembly	Targeted reporting of >200 well-characterized regions of known clinical significance with enriched SNP markers to provide an average resolution of ~20kb; outside these regions, average resolution is ~1 Mb
Lab H<br>N = 1	Illumina CytoSNP 850K	>800K markers (SNP)	GRCh37/hg19 assembly	Gains >500 kb and losses >200kb; Smaller CNVs only reported if they are likely to have clinical significance
Lab I<br>N = 34	Affymetrix Cytoscan HD	2.695 Million Targets (SNP + Copy Number)	GRCh37/hg19 assembly	Gains >2 Mb and losses >1 Mb; Smaller CNVs may be reported if pathogenic
Lab J<br>N = 1	UNK	UNK	UNK	UNK
Lab K<br>N = 2	UNK	UNK	UNK	>300 kb
Lab L<br>N = 1	Affymetrix Cytoscan HD	2.695 Million Targets (SNP + Copy Number)	GRCh37/hg19 assembly	UNK
Lab M<br>N = 1	GenomeDX v5	180K oligonucleotide probes (118K copy number and 66K SNP)	GRCh37/hg19 assembly	Copy number changes of >200 kb on average across the genome; between 500 bp and 15 kb in >200 targeted regions; do not report duplications <500 kb or deletions <250 kb outside of targeted areas
Lab M – Targeted<br>N = 1	UNK	60K probes (42K copy number, targeted to well-characterized microdeletion/microduplication	GRCh37/hg19 assembly	Detects CNVs of 5-25 kb in over 100 targeted regions and CNVs of >1.5 Mb elsewhere

		syndrome regions; 18K SNP genotype probes) Genotype analysis restricted to 6 chromosomes (6, 7, 11, 14, 15, 20)		
Lab N<br><br>N = 3	Affymetrix Cytoscan HD (targeted)<br><br>(SOMA)	2.695 Million Targets (SNP + Copy Number)	GRCh37/hg19 assembly	Gain or loss >100 kb within the targeted panel of clinically significant gene regions; Gain or loss outside the targeted regions with a contiguous dosage change across an interval of 1000 kb or greater, a density of 20 or greater probes per 100 kb segment and at least one OMIM annotated gene
Lab O<br><br>N = 6	Illumina CytoSNP 850K	>850K genome-wide markers	GRCh37/hg19 assembly	UNK
Lab P<br><br>N = 5	CGH + SNP microarray (Agilent)	180K	GRCh37/hg19 assembly	Lower limit of detection for CNVs is ~250 kb over the entire genome for detection of aberrations <10 kb possible in ISCA regions, however, copy number aberrations <10 kb will not be reported unless they correspond to pathogenic or likely pathogenic findings

Mb = megabse, kb = kilobase, SNP = single nucleotide polymorphism, CNV = copy number variant, OMIM = Online Mendelian Inheritance in Man
